# Macrophage Depletion Attenuates Extracellular Matrix Deposition and Ductular Reaction in a Mouse Model of Chronic Cholangiopathies

**DOI:** 10.1371/journal.pone.0162286

**Published:** 2016-09-12

**Authors:** Jan Best, Stefaan Verhulst, Wing-Kin Syn, Kimberly Lagaisse, Noemi van Hul, Femke Heindryckx, Jan-Peter Sowa, Liesbeth Peeters, Hans Van Vlierberghe, Isabelle A. Leclercq, Ali Canbay, Laurent Dollé, Leo A. van Grunsven

**Affiliations:** 1 Liver Cell Biology Laboratory, Department of Basic Biomedical Sciences, Vrije Universiteit Brussel (VUB), Brussel, Belgium; 2 Department of Gastroenterology and Hepatology, University Hospital, University Duisburg-Essen, Essen, Germany; 3 Foundation for Liver Research, The Institute of Hepatology, London, United Kingdom; 4 Division of Gastroenterology and Hepatology, Medical University of South Carolina, Charleston, South Carolina, United States of America; 5 Laboratory of Hepato-Gastroenterology, Institut de Recherche Expérimentale et Clinique (IREC), Université catholique de Louvain (UCL), Brussel, Belgium; 6 Department of Hepatology & Gastroenterology, Ghent University, Ghent, Belgium; 7 Section of Gastroenterology, Ralph H Johnson Veteran Affairs Medical Center, Charleston, South Carolina, United States of America; NCMLS, Radboud University Nijmegen Medical Center, NETHERLANDS

## Abstract

Chronic cholangiopathies, such as primary and secondary sclerosing cholangitis, are progressive disease entities, associated with periportal accumulation of inflammatory cells, encompassing monocytes and macrophages, peribiliary extracellular matrix (ECM) deposition and ductular reaction (DR). This study aimed to elucidate the relevance of macrophages in the progression of chronic cholangiopathies through macrophage depletion in a 3,5-diethoxycarbonyl-1,4-dihydrocollidine (DDC) mouse model. One group of mice received a single i.p. injection of Clodronate encapsulated liposomes (CLO^Lipo^) at day 7 of a 14 day DDC treatment, while control animals were co-treated with PBS^Lipo^ instead. Mice were sacrificed after 7 or respectively 14 days of treatment for immunohistochemical assessment of macrophage recruitment (F4/80), ECM deposition (Sirius Red, Laminin) and DR (CK19). Macrophage depletion during a 14 day DDC treatment resulted in a significant inhibition of ECM deposition. Porto-lobular migration patterns of laminin-rich ECM and ductular structures were significantly attenuated and a progression of DR was effectively inhibited by macrophage depletion. CLO^Lipo^ co-treatment resulted in a confined DR to portal regions without amorphous cell clusters. This study suggests that therapeutic options selectively directed towards macrophages might represent a feasible treatment for chronic cholestatic liver diseases.

## Introduction

Cholestasis is a pathologic condition where bile flow from the liver to the duodenum is impaired, clinically expressed by pruritus, jaundice, pale stool and dark urine. It can be attributed to two different basic distinctions [1]. Cholestasis can be related to parenchymal hepatic damage, associated with viral hepatitis or metabolic diseases. In the latter case disturbances in bile formation are caused by either genetic defects or are acquired in the context of medication or toxicity [2–4]. In contrast, during an obstructive type of cholestasis a mechanical blockage of the duct system is a result of various pathologic conditions. Among gallstones or malignancy, so called chronic cholangiopathies can also impair biliary drainage, the two representative disease conditions are primary sclerosing cholangitis (PSC) and primary biliary cirrhosis (PBC). PSC is characterized by inflammation of the bile ducts (cholangitis), encompassing peribiliary strictures and sclerosis due to fibrous scar formation, resulting in impaired bile flow and consecutive chronic liver injury culminating in liver cirrhosis [5]. In contrast to PSC, PBC is an autoimmune disease of the liver, characterized by slow progressive destruction of the small bile ducts of the liver, with the intralobular ducts and the Canals of Hering (intrahepatic ductules) affected early in the disease [6].

Cholangiopathies range among the leading causes of liver transplantation in paediatric and adult patients worldwide [7]. They can be triggered genetically such as Alagille´s syndrome and Cystic Fibrosis. Furthermore, various infectious conditions of bacterial, fungal, parasitic and viral origin can also induce cholangitis. While PSC and PBC are assumed to be mediated immunologically, its histological pendant, secondary sclerosing cholangitis (SSC)[8] is a result of obstruction or injury of the biliary tree [9]. Eventually, PSC and SSC, both very well characterized and clinically relevant representatives of chronic cholangiopathies lead to biliary fibrosis and cirrhosis [10]. Both diseases share common histopathological features including the presence of progressive damage leading to atrophy of medium- and large-size bile ducts. This is followed by compensatory cholangiocytic proliferation, referred to as ductular reaction (DR) [11], which is associated with periductular fibrosis and inflammation [12].

The 3,5-diethoxycarbonyl-1,4-dihydrocollidine (DDC) diet induces a cholangiopathy and is well validated to study the mechanisms of chronic cholangiopathies. DDC feeding leads to increased biliary porphyrin deposition associated with a pericholangitis, macrophage accumulation and activation of periductal myofibroblasts, eventually leading to a biliary type of fibrosis with an atypical ductular reaction [13].

Both human and DDC-induced murine cholangiopathies, are characterized by an “atypical” irregular proliferation of intrahepatic bile ducts, not only confined to portal areas but also sprouting into periportal and parenchymal regions, also known as a type II ductular reaction[14]. In affected patients, this is preceded by a type I reaction, regarded as an early stage of cholestatic liver disease, while type II refers to ductular metaplasia of liver cell plates, predominantly observed under chronic cholestatic conditions [15, 16]. We hypothesized that a persistent inflammatory response promotes progression from a typical biliary proliferation to an atypical biliary metaplasia and set out to investigate if inhibition of the inflammatory process would attenuate this process. To this end, we depleted macrophages by administration of liposome encapsulated Clodronate (CLO^Lipo^), as previously described by Van Rooijen *et al*. [17].

We report here that macrophage depletion in a murine model of chronic cholangiopathy results in a significant attenuation of DDC-induced changes within the portal and periportal micro-architecture. Macrophage depletion was associated with a more confined ductular reaction, resembling a DR type I[14]. In contrast, DDC treatment in mice without macrophage depletion induced an atypical ductular reaction, with sprouting of non-functional, poorly differentiated intrahepatic bile ductular structures, resembling a DR type II observed in patients suffering from late-stage cholangitis. These data suggest that macrophages sustain a persistent inflammatory response that promotes the transition from a type I to a type II DR.

## Results

### DDC diet induces biliary damage in mice

**Fig 1:**
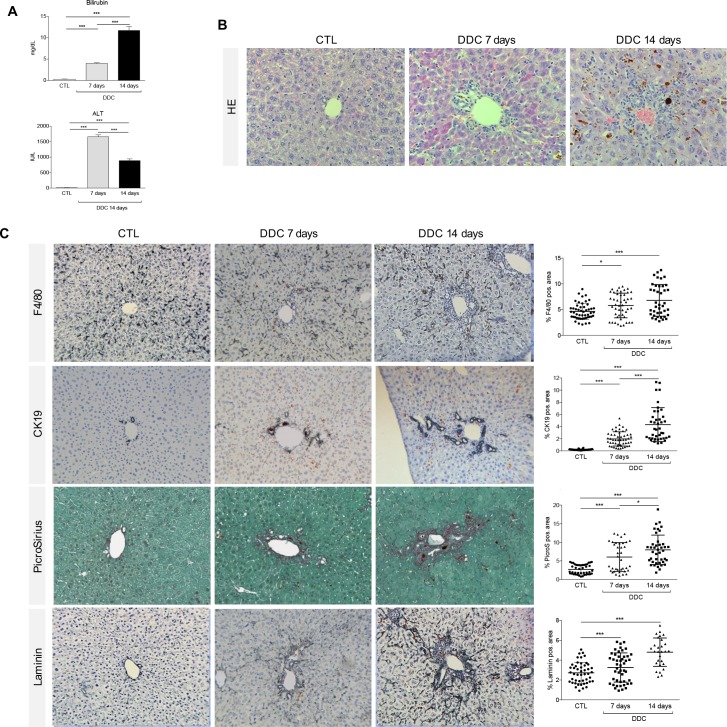
DDC diet induces biliary damage in mice. (A) Bilirubin and ALT serum levels were measured from control animals and after 7 and 14 days of DDC-diet with a clinical chemistry module. (B, C) Liver sections of control animals and mice subjected to DDC-Diet for 7 and 14 days were stained with HE, anti-F4/80 Ab, anti-CK19 Ab, Sirius Red and anti- Laminin Ab as described in material and methods. All images were taken in 40x original magnification. The percentage of F4/80-, CK19-, PicroSirius- and Laminin -positive area per field was assessed as described in detail in materials and methods section. All data are presented as mean ± SEM for n = 5/group, *p<0.05 and ***p<0.001.

In mice fed with the DDC diet for 14 days (see **S1 Fig** for the experimental setting), elevated serum levels of bilirubin, a marker of biliary injury and cholestasis in general, resemble the alterations described in human cholestatic liver diseases (**Fig 1A and 1B**). In these animals, DDC feeding induced cholangitis with increasing periductular fibrosis (Sirius Red positive collagen fibers), accumulation of macrophages (F4/80) and a pronounced DR (CK19^+^) over time (**Fig 1C**). In healthy mice, macrophages are randomly distributed in the liver parenchyma and accumulate after DDC treatment in periportal regions, inducing a local inflammatory response. In addition, there are only few CK19 positive bile ducts per portal tract in control mice, which markedly increase in size and number upon DDC treatment (**Fig 1C**). In DDC fed mice, these accumulating CK19 positive cells aggregate around the portal area organized in pseudoducts around a (pseudo) lumen. mRNA levels of F4/80, CK19, collagen-1, laminin (Lamc1) and α-smooth muscle actin (α-SMA, marker of hepatic stellate cell activation) support the histological findings (see **S2 Fig)**, as well as the morphometric analysis (**Fig 1C**) and suggest that this cascade of events starts with a clinically relevant liver injury, here assessed by increased ALT and bilirubin serum levels (**Fig 1A**). Onset of cholestasis (bilirubin increase) and upregulation of F4/80- and α-SMA-mRNA levels were already recorded at day 7 (**S2 Fig**), while a clear increase in collagen deposition, CK19-positive cells and laminin expression was detected at day 14.

### Macrophage depletion preserves a type I ductular reaction in DDC fed mice

Injection of liposomal clodronate (CLO^Lipo^) is a widely used tool to assess the impact of macrophage absence on liver injury. When applied in mouse models of hepatocyte injury, the biliary epithelial cells form bile duct-like structures instead of infiltrating the parenchyma to regenerate hepatocytes [18, 19]. No such experiments have been carried out in models of cholestatic injury. To investigate the impact of macrophage depletion on the DR induced by a DDC diet we first administered CLO^Lipo^ at the start of the DDC diet and analyzed the mice 7 days later. One injection of CLO^Lipo^ resulted in an almost complete absence of F4/80 positive cells, a strong reduction of the DR and less myofibroblast (αSMA) and ECM production in livers of DDC-treated mice (**S5 and S6 Figs)**. This suggests that macrophages are needed to induce the DR in this mouse model of cholestasis.

**Fig 2:**
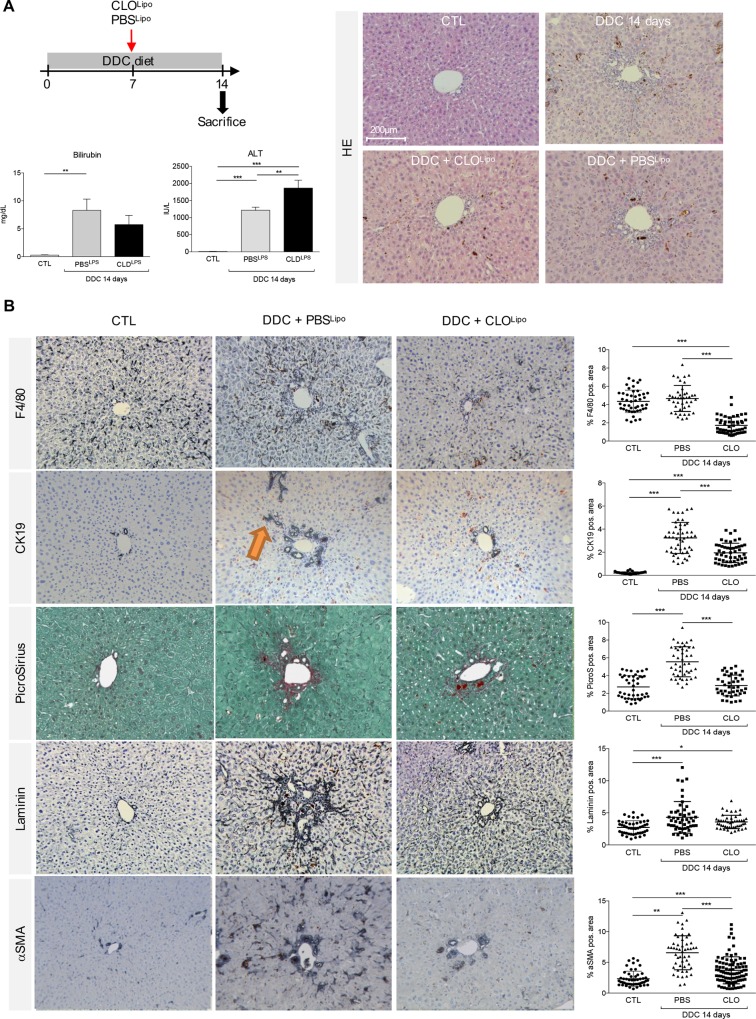
Macrophage depletion attenuates activation of myofibroblast like cells and preserves a type I ductular reaction in DDC fed mice. (A) Bilirubin and ALT serum levels were measured from control animals and mice subjected to a 14 day DDC diet in co-treatment with either CLO^Lipo^ or PBS^Lipo^ at experimental day 7. (B) Liver sections were stained with HE, anti-F4/80 Ab, anti-CK19 Ab, Sirius Red, anti-Laminin Ab and anti-alpha-Sma Ab as described in material and methods. All single stained images were taken in 20x, original magnification. Orange arrow indicate irregular CK19^+^ cell clusters migrating from the portal tract into parenchyma or porto-portal (Type II DR). The percentage of F4/80-, CK19-, Sirius red-, Laminin- and αSma- positive area per field was assessed as described in materials and methods. All data are presented as mean ± SEM for n = 5/group. *p<0.05, **p<0.01, ***p<0.001.

We then asked whether an already established DR could be influenced by macrophage depletion and employed a single injection of CLO^Lipo^ at day 7 of a 14d DDC treatment, PBS liposomes were used as a control (PBS^Lipo^). HE staining clearly visualized obstruction of bile ducts by porphyrin plugs during all scenarios of the 14 days DDC treatment (**Figs 1B and 2A**). No significant decrease in bilirubin levels was observed when DDC mice were treated with CLO^Lipo^, suggesting that the billiary injury was not affected. However, ALT levels suggest that co-treatment does result in more hepatotoxicity (**Fig 2A**). While in control mice, F4/80^+^ macrophages were scattered randomly throughout the liver parenchyma, in animals that received PBS^Lipo^ during the last 7 days of DDC treatment the F4/80^+^ cells showed a shift from the parenchyma to the periductular region of the portal tracts at 14 days of DDC treatment, whereas CLO^Lipo^ co-treatment induced an overall reduction of macrophage numbers (**Fig 2B**). Compared to PBS^Lipo^, co-treatment with CLO^Lipo^, resulted in an attenuated periductular and diminished porto-portal fibrosis at 14 days of DDC treatment. The overall amount of extracellular matrix (ECM) deposition, represented by Sirius Red and laminin positive area, was significantly reduced under CLO^Lipo^ co-treatment compared to co-administration of PBS^Lipo^ (**Fig 2B**). Histological findings were supported by morphometric analysis (**Fig 2B**) and mRNA level quantification (**S3 Fig**). Interestingly, a concomitant decrease of α-SMA and TGF-β-mRNA levels, as well as a reduced NTPD2-mRNA level (**S4 Fig**) was also observed, indicating an overall inhibition of myofibroblast formation in CLO^Lipo^ co-treated mice.

Mice subjected to 14 days of DDC diet **(Fig 1B)** and PBS^Lipo^ revealed clusters of CK19^+^ cells assembled in irregular ductule-like or circular structures. In addition, some CK19^+^ aggregates were budding from proliferative (pseudo)-ducts, elongating and radiating from the portal region into the lobule (orange arrow in **Fig 2B**). In general, CK19^+^ cells were found to form amorphous ductular structures with a superposition of cells irregular in morphology and size encircling a poorly defined lumen reminiscent of a type II DR. In contrast, co-treatment with CLO^Lipo^ led to a portal restriction, where the (pseudo)-ducts expanded along the portal vein instead of growing towards the lobule (**Fig 2B**). Parenchymal radiation by the formation of filamentous elongations as observed in the PBS^Lipo^ treated group was barely seen in the CLO^Lipo^ livers, further emphasizing the portal restriction resulting from macrophage depletion. The biliary structures were strictly organized in monolayer, ring-shaped structures, giving the impression of a type I DR.

### Macrophage depletion attenuates ECM deposition in DDC fed mice

**Fig 3:**
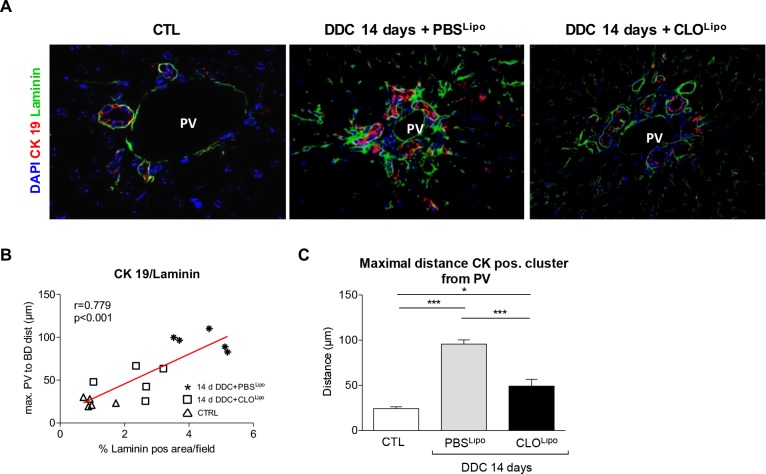
Overall amount of laminin matrix deposition and maximum migration distance of CK19 positive cell clusters is positively correlated. (A) Liver sections of control animals and mice subjected to a 14 day DDC-Diet in co-treatment with either CLO^Lipo^ or PBS^Lipo^ at experimental day 7, were co-stained with CK19/Laminin to assess the correlation between CK19^+^ cell clusters and ECM. (B) Positive correlation between overall amount of laminin matrix deposition. (C) Maximum migration distance of CK19^+^ cell clusters from the portal vicinity into the lobule. All stainings and quantifications were performed as described in materials and methods. All data are presented as mean ± SEM for n = 5/group, *p<0.05 and ***p<0.001.

Deposition of ECM is a prerequisite for CK19^+^ cells to migrate away from the portal vein [20]. Mice treated with PBS^Lipo^ during the last 7 days of DDC treatment exhibited laminin deposition, usually confined to the basal laminae and expanding from the perivascular and periductular vicinity into the lobule (**Figs 2B and 3A**). In contrast, in CLO^Lipo^ mice, laminin envelopes the proliferating biliary structures without radiating into the parenchyma. Co-staining for CK19 and laminin revealed newly arising ductular proliferations chaperoned by laminin structures in CLO^Lipo^ mice. This observation is strongly supported by a positive correlation between the increasing distances of CK19^+^ cell clusters from the center of a portal vein to the overall laminin positive area/field (**Fig 3B and 3C and S7 Fig**).

### Macrophages modulate the proliferative status of CK19^+^ cells

**Fig 4:**
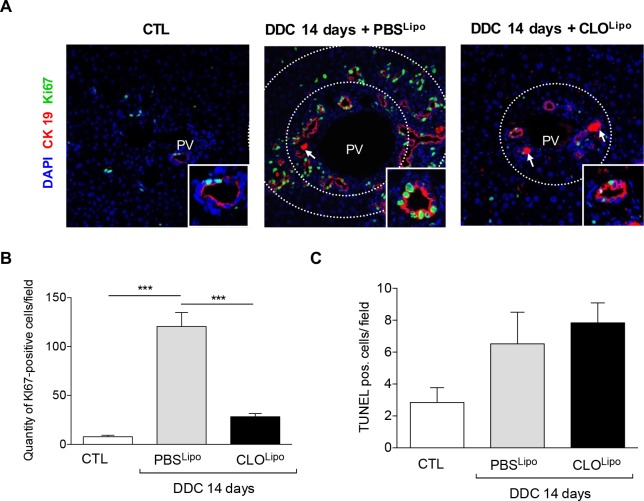
Under macrophage depletion proliferation of CK19 positive ductules in the portal region is inhibited. (A) Liver sections of the same mouse collective were co-stained with CK19/Ki67 to visualize the proliferation index. All images were taken in 40x original magnification. PV = Portal vein. White arrow indicating porphyrin deposition. (B) To quantify the proliferation index, the quantity of Ki 67-positive cells/field was determined. (C) To obtain the extent of apoptosis, TUNEL stainings were carried out (**S8 Fig)** and the amount of TUNEL pos. cells per field was quantified. All stainings and quantifications were performed as described in materials and methods. All data are presented as mean ± SEM for n = 5/group, ***p<0.001.

In the adult healthy liver, the majority of cholangiocytes is arrested in the G0 phase of the cell cycle [21, 22]. During the course of chronic cholestatic liver diseases, cholangiocytes proliferate to compensate for the anatomical and functional loss of injured biliary ducts [23]. While the number of CK19^+^ cells was clearly upregulated by DDC treatment, there was a significant reduction in the overall amount of CK19^+^ cells due to co-treatment with CLO^Lipo^ (**Fig 2B**). To investigate this further, we visualized the proliferation of CK19^+^ ductular structures by co-staining with Ki-67. After 14 days of DDC treatment there was a clear increase in Ki-67^+^/CK19^+^ cells (**Fig 4A and 4B**). In PBS^Lipo^ treated mice, the inner and outer zone of the portal vein contained numerous Ki-67^+^/CK19^+^ typical and atypical ductular structures. In CLO^Lipo^ treated mice, abundance of Ki-67^+^/CK19^+^ cells was lower, compared to their PBS^Lipo^ treated littermates. Interestingly, Ki-67^+^ cells were completely absent in ductules in the outer zone in mice that were treated with CLO^Lipo^ during the last half of the 14 days DDC treatment. This latter finding could not be attributed to increased apoptosis, since TUNEL staining demonstrated no significant difference in cell death (hepatocyte and cholangiocyte) between PBS or CLO treated DDC animals (**Fig 4C**).

## Discussion

**Fig 5:**
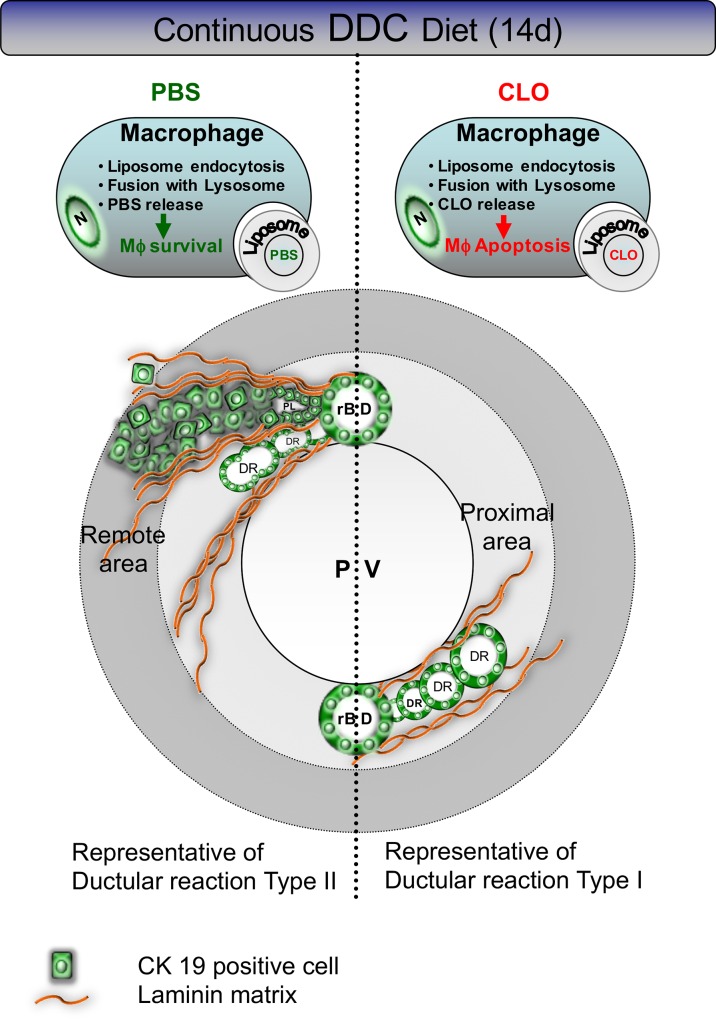
Schematic representation of the effect of macrophage depletion during DDC diet administration. DDC diet promotes a ductular reaction (DR) budding from resident bile ducts (rBD) chaperoned by laminin rich matrix. In PBS^Lipo^ co-treated mice (from day 7 onwards), a DR type II can be observed with CK19^+^ cells either forming amorphous bile ducts, clusters with pseudoluminae or rosettes, sprouting into the lobule. CLO^Lipo^ co-treatment (from day 7 onwards) preserves a type I DR, comprising confined ductular proliferates in proximity to the portal vein (PV). The newly budding CK19^+^ cells create more differentiated, bile ductular structures with regular concentric luminae.

It is clear that in chronic cholestatic liver diseases the primary target region of injury is the biliary epithelium, but the underlying pathophysiological mechanisms leading to the disease are still not entirely understood[21, 22]. Cholangiocellular proliferation to compensate for the loss of functional cholangiocytes in combination with varying degrees of inflammation and fibrosis are common in all cholangiopathies. During earlier stages, the “typical” compensatory hyperplasia of intrahepatic bile ducts is confined to portal spaces (type I DR) [24, 25], a progression to an “atypical” or irregular biliary proliferation can occur with ongoing disease. The latter includes proliferation of new intrahepatic bile ducts in direct proximity to the portal vein accompanied by a sprouting of functionally ineffective and amorphous ductular proliferates into the liver lobule (type II DR). To prevent disease progression, the development of a targeted treatment option is needed. We show that macrophage-depletion can prevent disease progression; i) activation of myofibroblast-like cells was attenuated, ii) ductular proliferates were confined to the portal region and newly arising CK19^+^ cell clusters remained more committed to a biliary phenotype. These findings suggest that macrophages play a key role in driving progression from a type I DR to a type II DR in this mouse model of advanced cholangiopathy (schematically represented in **Fig 5)**.

To investigate the interplay of different cell compartments during cholangiopathy progression, we used a xenobiotic-induced animal model resembling histological and clinical characteristics of chronic human cholangiopathic diseases. Human cholangiopathies are characterized by cholestasis, periportal inflammation and fibrosis accompanied by cholangiocyte proliferation [26]. Compared to bile duct ligation, which is commonly utilized as a surgical model of cholestatic liver diseases, the administration of a DDC diet comprises higher reproducibility and superior survival rates [27]. Consistent with the observations of Fickert *et al*. [13] we demonstrate that the DDC diet leads to increased biliary porphyrin deposition, pericholangitis, macrophage accumulation and activation of periductal myofibroblasts, eventually leading to a biliary type fibrosis with an atypical (type II) ductular reaction [13] and neo-vascularisation [28].

Macrophages have been shown to play a pivotal role in the progression of chronic cholangiopathies, predominantly by release of inflammatory cytokines, mediating stellate cell activation, with consecutive ECM deposition and fibrosis, finally culminating in secondary biliary cirrhosis. Previously, we have shown that depletion of immunocompetent cells significantly attenuated stellate cell activation[29] and that in a murine model of hepatic injury CLO^Lipo^ mediated macrophage depletion significantly attenuated ECM deposition[19]. Our current results show that macrophages also have this hepatic stellate cell inducing role during chronic cholangiopathies. Further investigation of these activated hepatic stellate cells reveals that they are the main laminin expressing cells in these 2 week DDC-injured mice when compared to hepatocytes, macrophages, liver sinusoidal endothelial cells and biliary epithelial cells (**S9 Fig**). In vitro, laminin determines the maintenance of the biliary cell phenotype[30]. We do not exclude that other cell types, e.g. portal myofibroblasts, might contribute to this increased laminin production and acknowledge that further studies are needed to determine whether in this injury setting laminin production also steers the biliary cell phenotype. In the healthy liver, laminins are predominantly expressed in the basal laminae around vessels and bile ducts. During liver injury they play a key role in tissue regeneration by modulating cell differentiation, migration and adhesion [31, 32]. In mice receiving DDC diet in combination with PBS^Lipo^, we observed that laminin-rich ECM expands from the basal laminae of vessels and bile ducts in a porto-portal and porto-central direction. The co-staining for CK19/laminin suggests that laminin creates a scaffold, embedding the CK19^+^ ductular structures and guiding migration of CK19^+^ cells into the lobule. This is supported by a significant correlation of the maximal migration distance of CK19^+^ cells to the overall amount of laminin deposition.

It is puzzling that in our experimental setting we see a clear inhibition of proliferation in the DDC and CLO^Lipo^ co-treated animals, while previous studies using similar liposomes in mice fed with a CDE diet (hepatocytic injury) did not result in a significant reduction of biliary epithelial cell proliferation [19]. The proposed underlying mechanism by which macrophages induce biliary epithelial cell proliferation was addressed over a decade ago; oval cell expansion induced by DDC was significantly reduced in wild-type mice with a blocking anti-TWEAK mAb as well as in Fn14-null mice (TWEAK receptor) [28]. These findings were subsequently confirmed in mice fed with a CDE diet using the same KO mice[33]. Finally, the Forbes lab showed that the TWEAK responsible for the biliary epithelial cell proliferation is most likely macrophage-deriven[34]. The latter finding supports our results in which depletion of the macrophages results in less proliferation, suggesting that the major source of TWEAK during a DDC-induced liver injury is indeed macrophages, while during a CDE-induced injury other cells might compensate. Important for potential therapeutic interventions using CLO^Lipo^ is that both resident macrophages (Kupffer cells) and infiltrating monocyte-derived macrophages can be depleted by these liposomes [35]. Further experiments using MCP-1/CCL2 Spiegelmers (mNOX-E36) [36], that specifically block recruitment of infiltrating monocytes, could reveal whether resident- or infiltrating macrophages should be targeted in progressive cholangiopathies.

In conclusion, persistent inflammation causes excessive ECM deposition in chronic cholestatic liver disease culminating in biliary cirrhosis. CLO^Lipo^-mediated macrophage depletion in our model could reduce this excessive ECM deposition and the DR progression suggesting that macrophage depletion might be a therapeutic option in progressive cholangiopathies.

## Material and Methods

### Animal experiments

All methods, experimental protocols and animal experiments ethics were carried out in accordance with the approved guidelines of the Vrije Universiteit Brussel (VUB, Belgium), conformed to European Guidelines for the Care and Use of Laboratory Animals. All animal experimental protocols were approved by the Ethical Committee of Animal Experimentation of the Vrije Universiteit Brussel (VUB, Belgium) (LA 123 02 12). Temperature, humidity and light-dark cycle (with a day light period from 07.00 a.m. to 19.00 p.m.) controlled conditions were maintained; 5 mice were allocated per cage and allowed food and water *ad libitum* and were routinely tested for background pathogens (QM Diagnostics, Nederland’s). After one week of acclimatization, 10-week-old male C57BL/6J mice (Charles River, Belgium) were randomly assigned to different experimental groups (see **S1 Fig**). Group I: mice were fed with a 3,5-diethioxycarbonyl-1,4-dihydrocollidine (0,1% wt/wt) (DDC, C14H21NO4, Sigma-Aldrich, 137030, Belgium) containing diet (C1000, Altromin, Lage, Germany) during a time course of 7 and 14 days. Mice used as control animals were fed with diet control (C1000, Altromin, Lage, Germany).

For macrophage depletion during DDC diet administration, mice in group II were co-treated with i.p. injections of CLO^Lipo^ in the test group (200μl/mouse) and in the control group with PBS encapsulated liposomes PBS^Lipo^ (200μl/mouse), respectively (at least 5 mice per group), as explained in **S1 Fig.** Liposomes were manufactured as described before [17] and the Clodronate concentration was 250mg/ml.

Mice were sacrificed under Pentobarbital anaesthesia (Nembutal^TM^, Ceva). A part of the caudate liver lobe was ligated with surgical silk, excised and snap-frozen for later isolation of RNA. Blood samples were taken from the inferior vena cava. For tissue fixation, we employed transcardial *in situ* perfusion with 4% formalin (10 min. at 7ml/min), preceded by a 5 min. PBS washing step for removal of the majority of blood cells. Livers were excised, collected in 4% formalin solution and embedded in paraffin (Leica TP1020). 4μm sections were cut (Microm, HM340E) and placed on silane coated slides (A3648, Sigma).

### Determination of serum parameters

Blood samples were centrifuged at 2,000g for 10 minutes and stored at -20°C. Serum levels of total bilirubin were determined with a clinical chemistry module (Spotchem EZ SP-4430, Axon Lab AG, Stuttgart, Germany).

### Histology and immunohistochemistry

#### Immunohistochemistry

4μm thick slides were deparaffinised and rehydrated. Endogenous peroxidase was blocked by immersion with 3% H_2_O_2_ in methanol for 20 min. Antigen retrieval was either heat mediated (Citrate buffer, PP20-0227, Prosan; pH 6, 95°C, 20 min, cooling at RT for 20 min) or by Proteinase K digestion (Dako, S3004, 10 min. at RT). To block nonspecific binding, slides were treated for 40 min. with 2% BSA (Sigma, A 2153) prior to incubation with the primary antibodies (1h at RT) or secondary antibodies (30min. at RT). See **S1 Table** for antibody characteristics. Between incubations, slides were washed with PBS/0.1% Tween for 3 times 5min. Peroxidase activity was detected by diaminobenzidine (MP Biomedicals,150835) or by Vector VIP (Vector Labs, SK-4600). Counterstaining of the slides was carried out either by Hematoxylin or by Methyl Green (Vector Labs, H-3402) according to the manufacturer instructions. Peroxidase stained histological sections were viewed on an Axioskop light microscope (Carl Zeiss, Munich, Germany) and pictures recorded using an Axiocam digital camera (Carl Zeiss). Fluorescent co-stainings (Laminin/CK19, Ki-67/CK19) were analyzed using a confocal fluorescent microscope (Zeiss LSM 710 NLO).

#### Picrosirius staining

To assess collagen deposition, 4μm paraffin sections were deparaffinised, rehydrated, and fixed with SUSA’s fixative for 1h and stained for 45min. with 0.1% Sirius Red F3BA/0.1% Fast Green FCF (both Sigma) in a saturated picric acid solution.

#### TUNEL Staining

Terminal deoxynucleotidyl transferase dUTP nick-end-labeling (TUNEL) staining was performed using the In Situ Cell Death Detection Kit, POD (Roche Diagnostics, Germany) according to the manufacturer’s instructions.

#### Morphometric quantification

For further morphometric quantification of F4/80, aSMA, Sirius Red, laminin and CK19 (at least 10 photographs/section of 20x magnification), pictures of respective slides were recorded using an Axiocam digital camera (Carl Zeiss). Automated quantification of the percentage of F4/80, αSMA, Sirius Red, Laminin and CK19 positive pixels/field was performed with ImageJ-Fiji software (http://fiji.sc/).

The algorithm for determination of the maximum distance from the center of a portal vein to the most remote corresponding bile duct and the normalisation of the overall CK19 positive area to the surface area of the corresponding portal vein are explained in detail in **S7 Fig**.

### mRNA analysis

Frozen mouse liver tissue was grinded to powder in liquid nitrogen and dissolved in lysis buffer containing 0.1% ß-Mercaptoethanol. Total RNA from tissue cultured cells was extracted using GeneJet^TM^ RNA purification kit (Fermentas, St. Leon-Rot, Germany). RNA was reverse-transcribed using RevertAid^TM^Premium Reverse Transcriptase (Fermentas) at 25°C for 10min., 30min. at 50°C and at 85°C for 5 min. Gene-specific primers and a Universal Probe Library probe were determined using Probe Finder software of Roche (https://www.roche-applied-science.com) and produced by Invitrogen and Roche (universal probe library, Mannheim, Germany), respectively (see **S2 Table**). Maxima® Probe qPCR Master Mix (Fermentas), 8 μl cDNA template, primer and probe were mixed to a final volume of 20μl and subjected to quantitative PCR (qPCR) in an ABI 7500 Real Time PCR system (Applied Biosystems, Foster City, CA, USA) using 18S ribosomal RNA for normalization. Each qPCR was performed by 40 cycles and melt curves were analyzed to unsure primer specificity. The fold change differences were determined using the comparative threshold cycle method.

### Statistical analysis

Data were analyzed using SPSS 17 (Statistical Package for Social Sciences, Chicago, IL, USA) licensed to the University of Essen, Germany. Frequencies, mean values (and standard errors of the mean) and proportions were reported when appropriate. Categorial data were compared using the chi-squared test and continuous data by the student’s t-test, or, if appropriate by nonparametric tests (ANOVA) and Tukey as an additional multiple comparison test. All p-values calculated were two-tailed; α<0.05 was considered significant for primary parameters with Bonferroni’s correction to control for type I error, if appropriate. Correlations between individual datasets were assessed by univariate analysis where applicable.

## Supporting Information

S1 Fig Experimental design and time course of DDC diet and administration of liposomes. (PDF)

S2 Fig Analysis of mRNA expression levels of F4/80, CK19, Collagen-I, Laminin and αSMA genes in DDC treated mice. Livers of control animals and mice subjected to a 7 or 14 day DDC-diet were collected. Hepatic mRNA expression of F4/80, CK19, Collagen-1, Laminin (Lamc1) and α-SMA (Acta2) was normalized to GAPDH mRNA as reference gene and expressed in relation to the mean value in untreated controls. All data are presented as mean ± SEM for n = 4/group, *p<0.05, **p<0.01, ***p<0.001.
(PDF)

S3 Fig Analysis of mRNA expression levels of F4/80, CK19, Collagen-I, Laminin and αSMA genes in DDC treated mice co-treated with PBS or Clodronate liposomes. Livers of control animals and mice subjected to a 14 day DDC-diet in co-treatment with either CLO^Lipo^ or PBS^Lipo^ at experimental day 7 were collected. Hepatic mRNA expression of F4/80, CK19, Collagen-1, Laminin (Lamc1) and α-SMA (Acta2) was normalized to GAPDH mRNA as reference gene and expressed in relation to the mean value in untreated controls. All data are presented as mean ± SEM for n = 4/group, *p<0.05, **p<0.01, ***p<0.001.
(PDF)

S4 Fig mRNA expression of profibrogenic genes in DDC treated mice. Hepatic mRNA expression of α-SMA (Acta2), TGF-β (markers of profibrogenic hepatic stellate cell activity) and NTPD2 expressed in relation to the mean value in untreated controls. All data are presented as mean ± SEM for n = 4/group, *p<0.05, **p<0.01, ***p<0.001.
(PDF)

S5 Fig Impact of Clodronate in a prophylactic setting. (A) Experimental setting. (B) ALT and Total bilirubin serum levels were measured from control animals and mice subjected to a 7 day DDC diet in co-treatment with either CLO^Lipo^ or PBS^Lipo^ from the beginning of the experiment. Liver sections of the same mice as mentioned above, were stained with HE (C), Picro-Sirius and for F4/80, CK19, Laminin, and α-Sma as described in material and methods (D). All single stained images were taken in 20x, original magnification. All data are presented as mean ± SEM for n = 5/group. *p<0.05, **p<0.01, ***p<0.001 compared to controls.
(PDF)

S6 Fig Morphometric quantification and analysis of mRNA expression of F4/80, CK19, Collagen-I, Laminin and αSMA genes in DDC treated mice co-treated with Clodronate. Livers of control animals and mice subjected to a 7 day DDC-diet in co-treatment with either CLO^Lipo^ or PBS^Lipo^ at the beginning of the experiments were collected. (A) Morphometric analysis for each corresponding marker presented in S6 Fig. The percentage of F4/80-, CK19-, Sirius red-, Laminin- and αSMA- positive area per field was assessed as described in detail in materials and methods section (B) Hepatic mRNA expression of F4/80, CK19, Collagen-1, Laminin (Lamc1) and α-SMA (Acta2) was normalized to GAPDH mRNA as reference gene and expressed in relation to the mean value in untreated controls. All data are presented as mean ± SEM for n = 4/group, *p<0.05, **p<0.01, ***p<0.001.
(PDF)

S7 Fig Quantification of CK19^+^ area/portal tract and determination of the most remote CK19^+^ cell cluster/cell from center of portal vein. To objectify the observation, that Clodronate treatment during DDC food administration results in a more confined localization of the CK19^+^ positive ductular proliferates to the corresponding portal vein, the following measurement algorithm has been developed: Utilizing Zeiss Axio Vision Rel. 4.8 software, the distance between the center of the portal vein and the corresponding most remote CK19^+^ positive cell of a ductular proliferation (max. PV to CK19^+^cell dist.) (porto-ductular distance) within a portal tract was measured. To normalize the measured porto-ductular distance to the diameter of the corresponding portal vein, the calculated radius r = [(meanD1; D2)/2] was subtracted from the measured total distance. Preferentially portal tracts with almost circular lumen PV were selected, longitudinally or tangentially cut PV were excluded from evaluation. A minimum of 5 Portal tracts per animal was evaluated.
(PDF)

S8 Fig Representative images of TUNEL stainings carried out on Control, DDC 14 days co-treated with CLO^Lipo^ or PBS^Lipo^. The quantification is presented in the main text.
(PDF)

S9 Fig Laminin is mainly expressed in activated HSCs. To identify the source of laminin in livers of DDC treated animals we isolated hepatocytes, liver sinusoidal endothelial cells (LSEC), F4/80 positive macrophages (Mφ), hepatic stellate cells (HSC) and biliary epithelial cells (BEC). (A) A schematic representation of the isolation procedure carried out according to Guimaraes et al. (1) and gene expression analysis of Laminin (Lamc1) was performed on all different cell types. Briefly, we dissociate the liver using pronase and collagenase to obtain a single cell suspension. A centrifugation of 50g was performed to dissociate the non-parenchymal fraction from the hepatocytes. Hepatocytes were purified using a percoll gradient centrifugation step. We blocked the non-parenchymal fraction using bovine serum albumin for 10 min and incubated cells with the indicated antibodies for 15 min. After adding propidium iodide we used fluorescent activated cell sorting to isolate LSECs (CD32^+^ F4/80^-^ UV^-^ PI^-^), macrophages (F4/80^+^ CD32^-^ UV^-^ PI^-^), HSCs (UV^+^ CD32^-^ F4/80^-^ PI^-^) and BECs (EpCAM^+^, CD45^-^, UV^-^, PI^-^). (B) Purity control of the isolated cells using gene expression analysis for Cyp3a11(Hepatocytes), Desmin (HSC), F4/80 (macrophages), Stab2 (LSEC), Epcam (BEC) and αSMA (Acta2: activated HSCs) on the different celltypes. Gapdh is used as a reference gene.
(PDF)

S1 Table Antibodies used for immunofluorescence (IF), immunohistochemistry *(IHC)* or FACS. (PDF)

S2 Table Oligonucleotides used for RT-qPCR. (PDF)

## References

[1] Wagner M, Trauner M. Recent advances in understanding and managing cholestasis. F1000Research. 2016;5 10.12688/f1000research.8012.1 27134744 PMC4841200

[2] Brown AC. Liver toxicity related to herbs and dietary supplements: Online table of case reports. Part 3 of 6. Food and chemical toxicology: an international journal published for the British Industrial Biological Research Association. 2016 Epub 2016/07/13. 10.1016/j.fct.2016.07.001 . 27402097

[3] Lazaridis KN, LaRusso NF. The Cholangiopathies. Mayo Clin Proc. 2015;90(6):791–800. Epub 2015/05/11. 10.1016/j.mayocp.2015.03.017 25957621 PMC4533104

[4] Hirschfield GM. Genetic determinants of cholestasis. Clin Liver Dis. 2013;17(2):147–59. Epub 2013/04/02. 10.1016/j.cld.2012.12.002 . 23540495

[5] Nayagam JS, Pereira SP, Devlin J, Harrison PM, Joshi D. Controversies in the management of primary sclerosing cholangitis. World J Hepatol. 2016;8(5):265–72. Epub 2016/03/01. 10.4254/wjh.v8.i5.265 26925200 PMC4757649

[6] Mousa HS, Carbone M, Malinverno F, Ronca V, Gershwin ME, Invernizzi P. Novel therapeutics for primary biliary cholangitis: Toward a disease-stage-based approach. Autoimmunity reviews. 2016 10.1016/j.autrev.2016.07.003 . 27393766

[7] Alpini G, McGill JM, Larusso NF. The pathobiology of biliary epithelia. Hepatology. 2002;35(5):1256–68. Epub 2002/05/01. doi: S0270913902242184 [pii] 10.1053/jhep.2002.33541 . 11981776

[8] Strazzabosco M, Fabris L. Development of the bile ducts: essentials for the clinical hepatologist. J Hepatol. 2012;56(5):1159–70. Epub 2012/01/17. 10.1016/j.jhep.2011.09.022 S0168-8278(12)00037-2 [pii]. 22245898 PMC3328609

[9] Lazaridis KN. Sclerosing cholangitis epidemiology and etiology. J Gastrointest Surg. 2008;12(3):417–9. Epub 2007/10/25. 10.1007/s11605-007-0344-3 . 17957439

[10] Mendes F, Lindor KD. Primary sclerosing cholangitis: overview and update. Nat Rev Gastroenterol Hepatol. 2010;7(11):611–9. Epub 2010/10/13. doi: nrgastro.2010.155 [pii] 10.1038/nrgastro.2010.155 . 20938459

[11] Popper H, Kent G, Stein R. Ductular cell reaction in the liver in hepatic injury. J Mt Sinai Hosp N Y. 1957;24(5):551–6. Epub 1957/09/01. . 13476145

[12] Scheuer PJ. Ludwig Symposium on biliary disorders—part II. Pathologic features and evolution of primary biliary cirrhosis and primary sclerosing cholangitis. Mayo Clin Proc. 1998;73(2):179–83. Epub 1998/02/24. . 9473003 10.4065/73.2.179

[13] Fickert P, Stoger U, Fuchsbichler A, Moustafa T, Marschall HU, Weiglein AH, et al A new xenobiotic-induced mouse model of sclerosing cholangitis and biliary fibrosis. Am J Pathol. 2007;171(2):525–36. Epub 2007/06/30. 10.2353/ajpath.2007.061133 17600122 PMC1934539

[14] Priester S, Wise C, Glaser SS. Involvement of cholangiocyte proliferation in biliary fibrosis. World J Gastrointest Pathophysiol. 2010;1(2):30–7. Epub 2011/05/25. 10.4291/wjgp.v1.i2.30 21607140 PMC3097945

[15] Desmet VJ. The amazing universe of hepatic microstructure. Hepatology. 2009;50(2):333–44. Epub 2009/07/31. 10.1002/hep.23152 . 19642165

[16] Svegliati-Baroni G, De Minicis S, Marzioni M. Hepatic fibrogenesis in response to chronic liver injury: novel insights on the role of cell-to-cell interaction and transition. Liver Int. 2008;28(8):1052–64. Epub 2008/09/12. doi: LIV1825 [pii] 10.1111/j.1478-3231.2008.01825.x . 18783548

[17] Van Rooijen N, Sanders A. Liposome mediated depletion of macrophages: mechanism of action, preparation of liposomes and applications. Journal of immunological methods. 1994;174(1–2):83–93. Epub 1994/09/14. . 8083541 10.1016/0022-1759(94)90012-4

[18] Boulter L, Govaere O, Bird TG, Radulescu S, Ramachandran P, Pellicoro A, et al Macrophage-derived Wnt opposes Notch signaling to specify hepatic progenitor cell fate in chronic liver disease. Nat Med. 2012;18(4):572–9. Epub 2012/03/06. 10.1038/nm.2667 22388089 PMC3364717

[19] Van Hul N, Lanthier N, Espanol Suner R, Abarca Quinones J, van Rooijen N, Leclercq I. Kupffer cells influence parenchymal invasion and phenotypic orientation, but not the proliferation, of liver progenitor cells in a murine model of liver injury. Am J Pathol. 2011;179(4):1839–50. Epub 2011/08/23. 10.1016/j.ajpath.2011.06.042 21854752 PMC3181378

[20] Van Hul NK, Abarca-Quinones J, Sempoux C, Horsmans Y, Leclercq IA. Relation between liver progenitor cell expansion and extracellular matrix deposition in a CDE-induced murine model of chronic liver injury. Hepatology. 2009;49(5):1625–35. 10.1002/hep.22820 . 19296469

[21] Alpini G, Glaser SS, Ueno Y, Pham L, Podila PV, Caligiuri A, et al Heterogeneity of the proliferative capacity of rat cholangiocytes after bile duct ligation. Am J Physiol. 1998;274(4 Pt 1):G767–75. Epub 1998/05/12. . 9575860 10.1152/ajpgi.1998.274.4.G767

[22] Lesage G, Glaser SS, Gubba S, Robertson WE, Phinizy JL, Lasater J, et al Regrowth of the rat biliary tree after 70% partial hepatectomy is coupled to increased secretin-induced ductal secretion. Gastroenterology. 1996;111(6):1633–44. Epub 1996/12/01. doi: S0016-5085(96)70027-6 [pii]. . 8942744 10.1016/s0016-5085(96)70027-6

[23] Bogert PT, LaRusso NF. Cholangiocyte biology. Curr Opin Gastroenterol. 2007;23(3):299–305. Epub 2007/04/07. 10.1097/MOG.0b013e3280b079fb 00001574-200705000-00013 [pii]. . 17414846

[24] Alvaro D, Mancino MG, Glaser S, Gaudio E, Marzioni M, Francis H, et al Proliferating cholangiocytes: a neuroendocrine compartment in the diseased liver. Gastroenterology. 2007;132(1):415–31. Epub 2007/01/24. doi: S0016-5085(06)01666-0 [pii] 10.1053/j.gastro.2006.07.023 . 17241889

[25] Alvaro D, Gigliozzi A, Attili AF. Regulation and deregulation of cholangiocyte proliferation. J Hepatol. 2000;33(2):333–40. Epub 2000/08/22. doi: S0168-8278(00)80377-3 [pii]. . 10952254 10.1016/s0168-8278(00)80377-3

[26] Rust C, Gores GJ. Apoptosis and liver disease. Am J Med. 2000;108(7):567–74. Epub 2000/05/12. doi: S0002-9343(00)00370-3 [pii]. . 10806286 10.1016/s0002-9343(00)00370-3

[27] Chang ML, Yeh CT, Chang PY, Chen JC. Comparison of murine cirrhosis models induced by hepatotoxin administration and common bile duct ligation. World J Gastroenterol. 2005;11(27):4167–72. Epub 2005/07/15. . 16015684 10.3748/wjg.v11.i27.4167 PMC4615437

[28] Jakubowski A, Ambrose C, Parr M, Lincecum JM, Wang MZ, Zheng TS, et al TWEAK induces liver progenitor cell proliferation. J Clin Invest. 2005;115(9):2330–40. 10.1172/JCI23486 16110324 PMC1187931

[29] Canbay A, Feldstein AE, Higuchi H, Werneburg N, Grambihler A, Bronk SF, et al Kupffer cell engulfment of apoptotic bodies stimulates death ligand and cytokine expression. Hepatology. 2003;38(5):1188–98. 10.1053/jhep.2003.50472 . 14578857

[30] Lorenzini S, Bird TG, Boulter L, Bellamy C, Samuel K, Aucott R, et al Characterisation of a stereotypical cellular and extracellular adult liver progenitor cell niche in rodents and diseased human liver. Gut. 2010;59(5):645–54. Epub 2010/04/30. 10.1136/gut.2009.182345 20427399 PMC3034133

[31] Timpl R, Rohde H, Robey PG, Rennard SI, Foidart JM, Martin GR. Laminin—a glycoprotein from basement membranes. J Biol Chem. 1979;254(19):9933–7. Epub 1979/10/10. . 114518

[32] Sasaki T, Fassler R, Hohenester E. Laminin: the crux of basement membrane assembly. J Cell Biol. 2004;164(7):959–63. Epub 2004/03/24. 10.1083/jcb.200401058 jcb.200401058 [pii]. . 15037599 PMC2172061

[33] Tirnitz-Parker JEE, Viebahn CS, Jakubowski A, Klopcic BRS, Olynyk JK, Yeoh GCT, et al Tumor necrosis factor-like weak inducer of apoptosis is a mitogen for liver progenitor cells. Hepatology. 2010;52(1):291–302. 10.1002/hep.23663 20578156

[34] Bird TG, Lu WY, Boulter L, Gordon-Keylock S, Ridgway RA, Williams MJ, et al Bone marrow injection stimulates hepatic ductular reactions in the absence of injury via macrophage-mediated TWEAK signaling. Proceedings of the National Academy of Sciences. 2013 10.1073/pnas.1302168110 PMC3631632 23576749

[35] van Rooijen N, Hendrikx E. Liposomes for specific depletion of macrophages from organs and tissues. Methods Mol Biol. 2010;605:189–203. Epub 2010/01/15. 10.1007/978-1-60327-360-2_13 . 20072882

[36] Baeck C, Wehr A, Karlmark KR, Heymann F, Vucur M, Gassler N, et al Pharmacological inhibition of the chemokine CCL2 (MCP-1) diminishes liver macrophage infiltration and steatohepatitis in chronic hepatic injury. Gut. 2011;61(3):416–26. 10.1136/gutjnl-2011-300304 21813474

