# Supplementary material for: Macrophage Depletion Attenuates Extracellular Matrix Deposition and Ductular Reaction in a Mouse Model of Chronic Cholangiopathies
Source: PLoS One. 2016 Sep 12;11(9):e0162286. doi: 10.1371/journal.pone.0162286 (PMC5019458; doi:10.1371/journal.pone.0162286)
Supplement: S1 Fig — (PDF) [file pone.0162286.s001.pdf]

Animal experiments

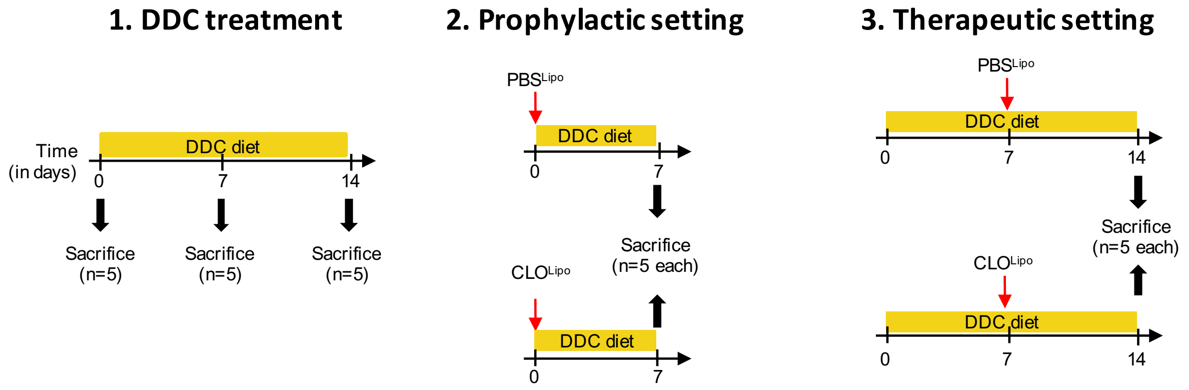

Supplementary Figure S1: Experimental design and time course of DDC diet and administration of liposomes.
