# Supplementary material for: Macrophage Depletion Attenuates Extracellular Matrix Deposition and Ductular Reaction in a Mouse Model of Chronic Cholangiopathies
Source: PLoS One. 2016 Sep 12;11(9):e0162286. doi: 10.1371/journal.pone.0162286 (PMC5019458; doi:10.1371/journal.pone.0162286)
Supplement: S2 Fig — Livers of control animals and mice subjected to a 7 or 14 day DDC-diet were collected. Hepatic mRNA expression of F4/80, CK19, Collagen-1, Laminin (Lamc1) and α-SMA (Acta2) was normalized to GAPDH mRNA as reference gene and expressed in relation to the mean value in untreated controls. All data are presented as mean ± SEM for n = 4/group, *p<0.05, **p<0.01, ***p<0.001. (PDF) [file pone.0162286.s002.pdf]

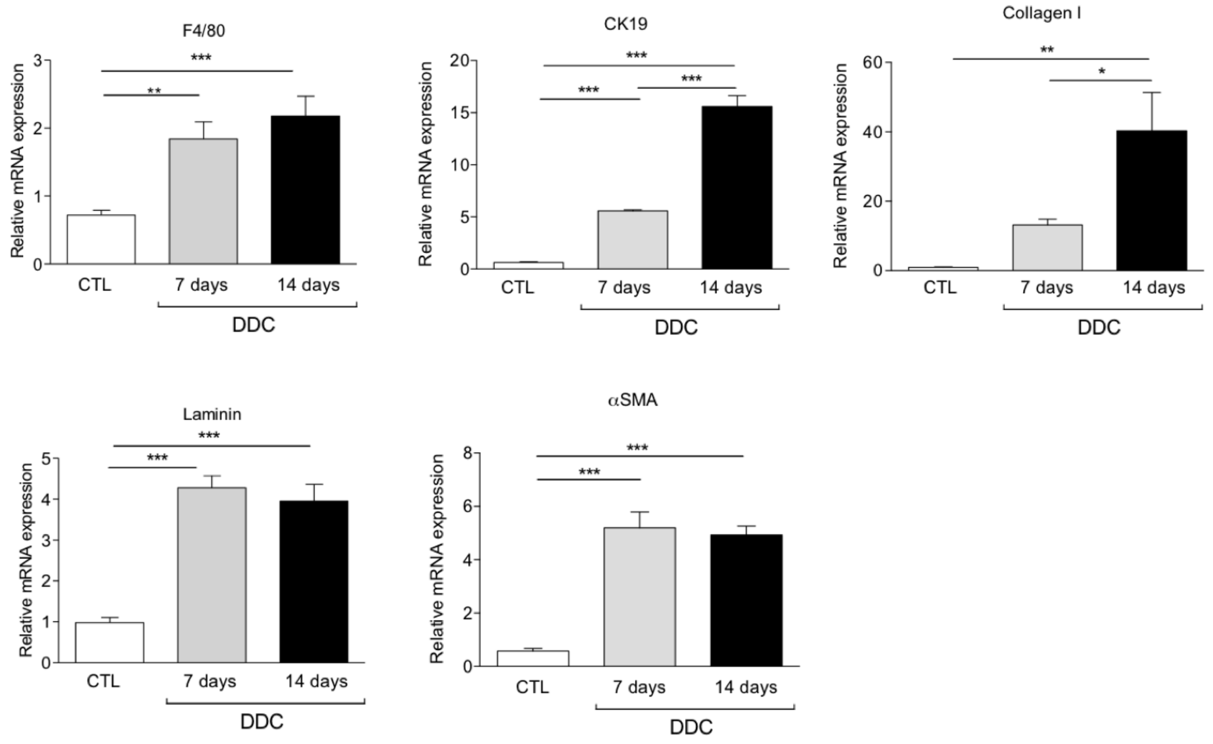

Supplementary Figure S2: Analysis of mRNA expression levels of F4/80, CK19, Collagen-I, Laminin and αSMA genes in DDC treated mice. Livers of control animals and mice subjected to a 7 or 14 day DDC-diet were collected. Hepatic mRNA expression of F4/80, CK19, Collagen-1, laminin and α-SMA was normalized to GAPDH mRNA as reference gene and expressed in relation to the mean value in untreated controls. All data are presented as mean ± SEM for n=4/group, *p<0.05, **p<0.01, ***p<0.001.
