# Supplementary material for: Macrophage Depletion Attenuates Extracellular Matrix Deposition and Ductular Reaction in a Mouse Model of Chronic Cholangiopathies
Source: PLoS One. 2016 Sep 12;11(9):e0162286. doi: 10.1371/journal.pone.0162286 (PMC5019458; doi:10.1371/journal.pone.0162286)
Supplement: S4 Fig — Hepatic mRNA expression of α-SMA (Acta2), TGF-β (markers of profibrogenic hepatic stellate cell activity) and NTPD2 expressed in relation to the mean value in untreated controls. All data are presented as mean ± SEM for n = 4/group, *p<0.05, **p<0.01, ***p<0.001. (PDF) [file pone.0162286.s004.pdf]

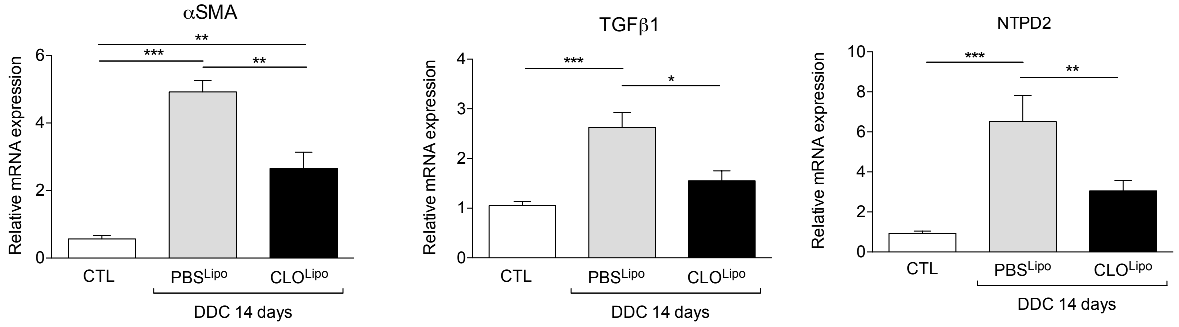

Supplementary Figure S4: mRNA expression of profibrogenic genes in DDC treated mice. Hepatic mRNA expression of α-SMA, TGF-β (markers of profibrogenic hepatic stellate cell activity) and NTPD2 expressed in relation to the mean value in untreated controls. All data are presented as mean ± SEM for n=4/group, *p<0.05, **p<0.01, ***p<0.001.
