# Supplementary material for: Macrophage Depletion Attenuates Extracellular Matrix Deposition and Ductular Reaction in a Mouse Model of Chronic Cholangiopathies
Source: PLoS One. 2016 Sep 12;11(9):e0162286. doi: 10.1371/journal.pone.0162286 (PMC5019458; doi:10.1371/journal.pone.0162286)
Supplement: S5 Fig — (A) Experimental setting. (B) ALT and Total bilirubin serum levels were measured from control animals and mice subjected to a 7 day DDC diet in co-treatment with either CLOLipo or PBSLipo from the beginning of the experiment. Liver sections of the same mice as mentioned above, were stained with HE (C), Picro-Sirius and for F4/80, CK19, Laminin, and α-Sma as described in material and methods (D). All single stained images were taken in 20x, original magnification. All data are presented as mean ± SEM for n = 5/group. *p<0.05, **p<0.01, ***p<0.001 compared to controls. (PDF) [file pone.0162286.s005.pdf]

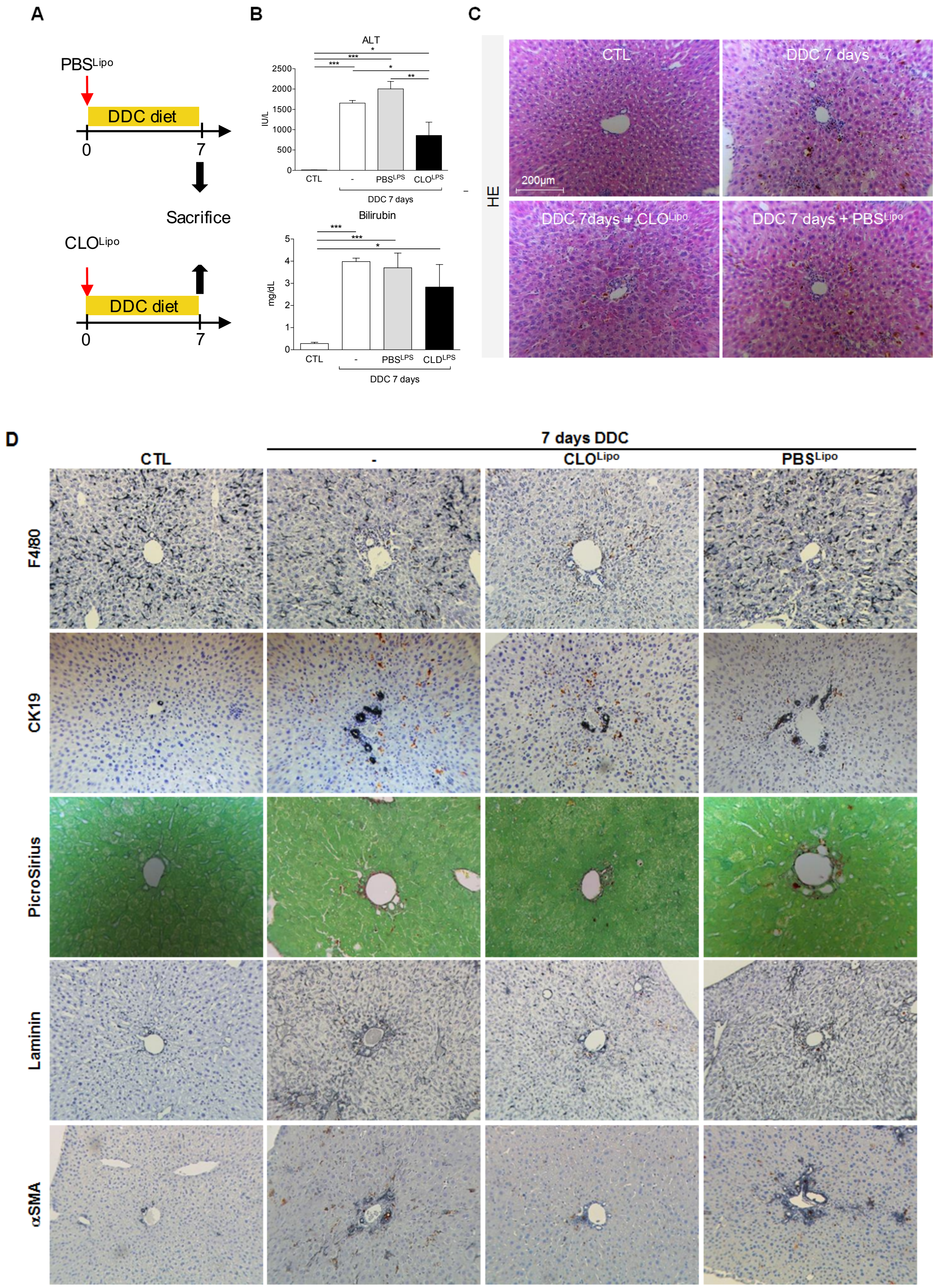

Supplementary Figure S5: Impact of Clodronate in a prophylactic setting. (A) Experimental setting. (B) ALT and Total bilirubin serum levels were measured from control animals and mice subjected to a 7 day DDC diet in co-treatment with either CLO^{Lipo} or PBS^{Lipo} from the beginning of the experiment. Liver sections of the same mice as mentioned above, were stained with HE (C), Picro-Sirius and for F4/80, CK19, Laminin, and α-Sma as described in material and methods (D). All single stained images were taken in 20x, original magnification. All data are presented as mean ± SEM for n=5/group. $^{*}p<0.05$, $^{**}p<0.01$, $^{***}p<0.001$ compared to controls.
