# Supplementary material for: Macrophage Depletion Attenuates Extracellular Matrix Deposition and Ductular Reaction in a Mouse Model of Chronic Cholangiopathies
Source: PLoS One. 2016 Sep 12;11(9):e0162286. doi: 10.1371/journal.pone.0162286 (PMC5019458; doi:10.1371/journal.pone.0162286)
Supplement: S7 Fig — To objectify the observation, that Clodronate treatment during DDC food administration results in a more confined localization of the CK19+ positive ductular proliferates to the corresponding portal vein, the following measurement algorithm has been developed: Utilizing Zeiss Axio Vision Rel. 4.8 software, the distance between the center of the portal vein and the corresponding most remote CK19+ positive cell of a ductular proliferation (max. PV to CK19+cell dist.) (porto-ductular distance) within a portal tract was measured. To normalize the measured porto-ductular distance to the diameter of the corresponding portal vein, the calculated radius r = [(meanD1; D2)/2] was subtracted from the measured total distance. Preferentially portal tracts with almost circular lumen PV were selected, longitudinally or tangentially cut PV were excluded from evaluation. A minimum of 5 Portal tracts per animal was evaluated. (PDF) [file pone.0162286.s007.pdf]

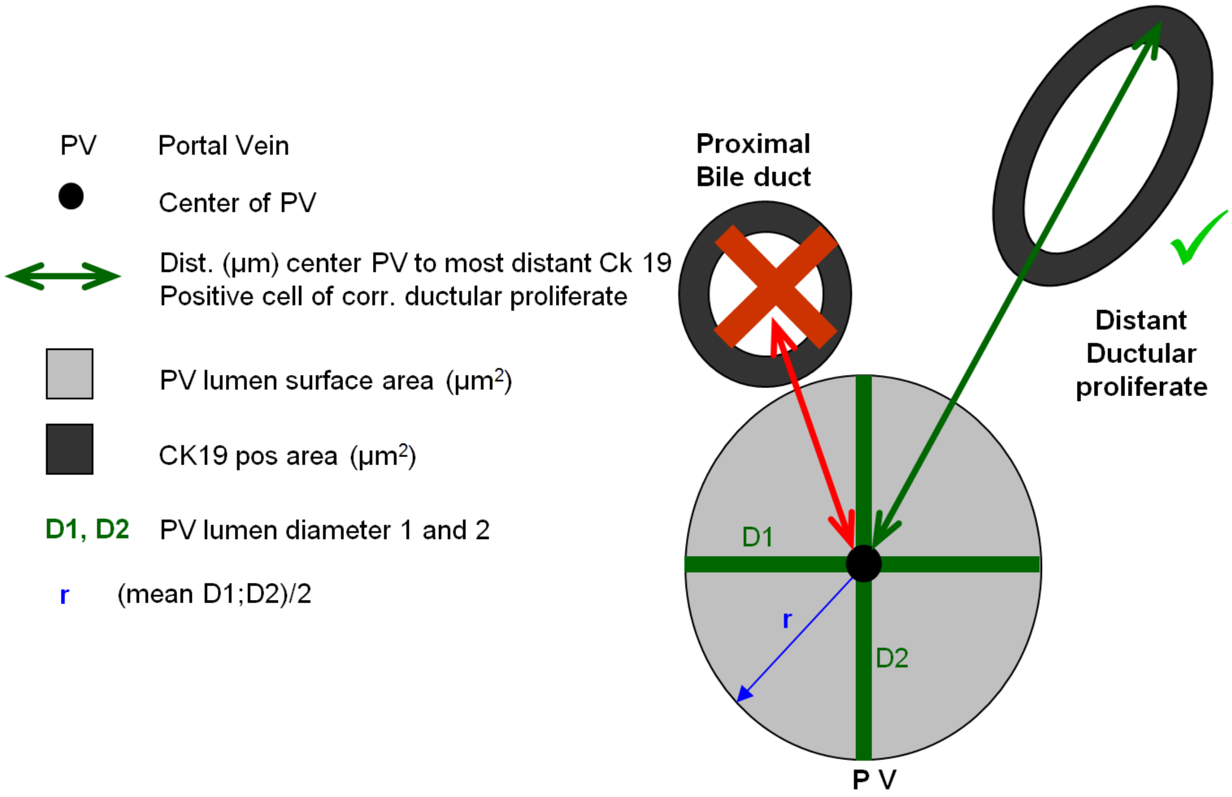

Supplementary Figure S7: Quantification of CK19$^+$ area/portal tract and determination of the most remote CK 19$^+$ cell cluster/cell from center of portal vein. To objectify the observation, that Clodronate treatment during DDC food administration results in a more confined localization of the CK19$^+$ positive ductular proliferates to the corresponding portal vein, the following measurement algorithm has been developed: Utilizing Zeiss Axio Vision Rel. 4.8 software, the distance between the center of the portal vein and the corresponding most remote CK19$^+$ positive cell of a ductular proliferation (max. PV to CK19$^+$cell dist.) (porto-ductular distance) within a portal tract was measured. To normalize the measured porto-ductular distance to the diameter of the corresponding portal vein, the calculated radius r=[(meanD1; D2)/2] was subtracted from the measured total distance. Preferentially portal tracts with almost circular lumen PV were selected, longitudinally or tangentially cut PV were excluded from evaluation. A minimum of 5 Portal tracts per animal was evaluated.
