# Supplementary material for: Macrophage Depletion Attenuates Extracellular Matrix Deposition and Ductular Reaction in a Mouse Model of Chronic Cholangiopathies
Source: PLoS One. 2016 Sep 12;11(9):e0162286. doi: 10.1371/journal.pone.0162286 (PMC5019458; doi:10.1371/journal.pone.0162286)
Supplement: S8 Fig — The quantification is presented in the main text. (PDF) [file pone.0162286.s008.pdf]

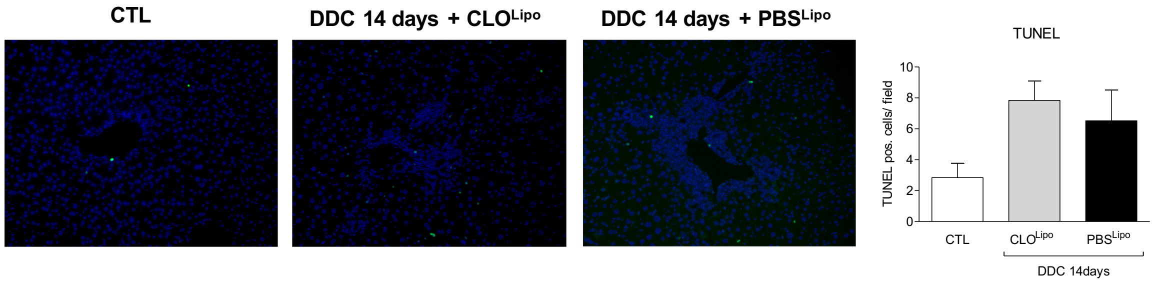

Supplementary Figure S8: Representative images of TUNEL stainings carried out on Control, DDC 14 days co-treated with CLO^{Lipo} or PBS^{Lipo}. The quantification is presented in the main text.
