# Supplementary material for: Macrophage Depletion Attenuates Extracellular Matrix Deposition and Ductular Reaction in a Mouse Model of Chronic Cholangiopathies
Source: PLoS One. 2016 Sep 12;11(9):e0162286. doi: 10.1371/journal.pone.0162286 (PMC5019458; doi:10.1371/journal.pone.0162286)
Supplement: S9 Fig — To identify the source of laminin in livers of DDC treated animals we isolated hepatocytes, liver sinusoidal endothelial cells (LSEC), F4/80 positive macrophages (Mφ), hepatic stellate cells (HSC) and biliary epithelial cells (BEC). (A) A schematic representation of the isolation procedure carried out according to Guimaraes et al. (1) and gene expression analysis of Laminin (Lamc1) was performed on all different cell types. Briefly, we dissociate the liver using pronase and collagenase to obtain a single cell suspension. A centrifugation of 50g was performed to dissociate the non-parenchymal fraction from the hepatocytes. Hepatocytes were purified using a percoll gradient centrifugation step. We blocked the non-parenchymal fraction using bovine serum albumin for 10 min and incubated cells with the indicated antibodies for 15 min. After adding propidium iodide we used fluorescent activated cell sorting to isolate LSECs (CD32+ F4/80- UV- PI-), macrophages (F4/80+ CD32- UV- PI-), HSCs (UV+ CD32- F4/80- PI-) and BECs (EpCAM+, CD45-, UV-, PI-). (B) Purity control of the isolated cells using gene expression analysis for Cyp3a11(Hepatocytes), Desmin (HSC), F4/80 (macrophages), Stab2 (LSEC), Epcam (BEC) and αSMA (Acta2: activated HSCs) on the different celltypes. Gapdh is used as a reference gene. (PDF) [file pone.0162286.s009.pdf]

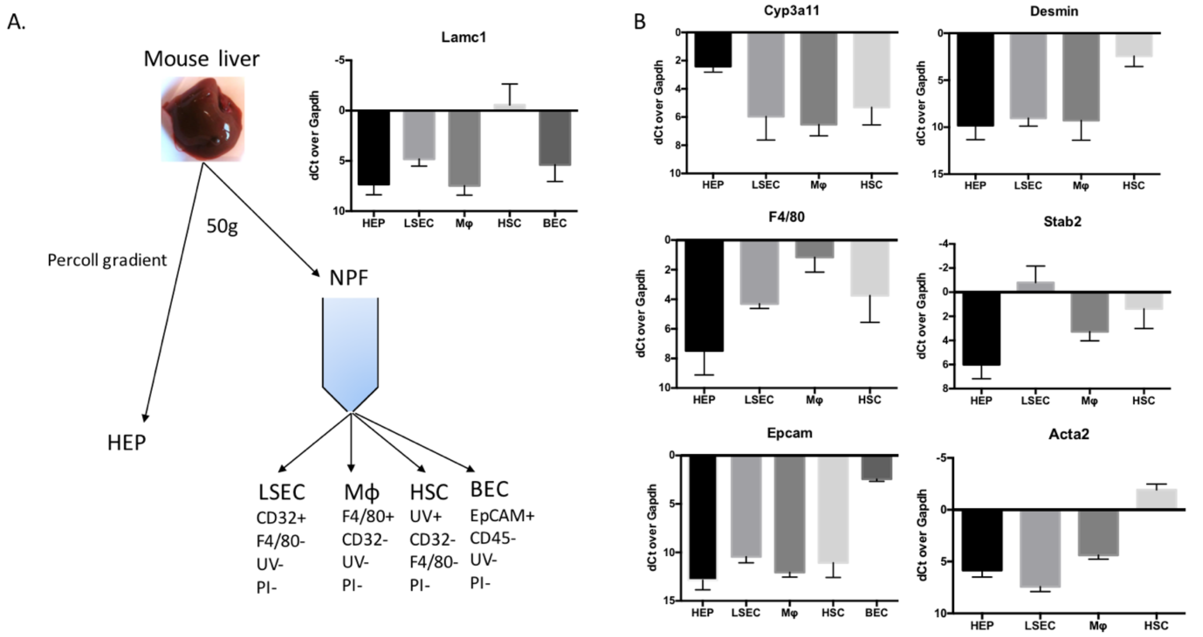

Supplementary Figure S9. Laminin is mainly expressed in activated HSCs. To identify the source of laminin in livers of DDC treated animals we isolated hepatocytes, liver sinusoidal endothelial cells (LSEC), F4/80 positive macrophages (Mφ), hepatic stellate cells (HSC) and biliary epithelial cells (BEC). (A) A schematic representation of the isolation procedure carried out according to Guimaraes et al. [1] and gene expression analysis of Lamc1 was performed on all different cell types. Briefly, we dissociate the liver using pronase and collagenase to obtain a single cell suspension. A centrifugation of 50g was performed to dissociate the non-parenchymal fraction from the hepatocytes. Hepatocytes were purified using a percoll gradient centrifugation step. We blocked the non-parenchymal fraction using bovine serum albumin for 10 min and incubated cells with the indicated antibodies for 15 min. After adding propidium iodide we used fluorescent activated cell sorting to isolate LSECs ($CD32^+$ $F4/80^-$ UV^- PI^-), macrophages ($F4/80^+$ $CD32^-$ UV^- PI^-), HSCs (UV^+ $CD32^-$ $F4/80^-$ PI^-) and BECs ($EpCAM^+$, $CD45^-$, UV^-, PI^-). (B) Purity control of the isolated cells using gene expression analysis for Cyp3a11(Hepatocytes), Desmin (HSC), F4/80 (macrophages), Stab2 (LSEC), Epcam (BEC) and Acta2 (activated HSCs) on the different celltypes. Gapdh is used as a reference gene.

1. Guimaraes EL, Stradiot L, Mannaerts I, Schroyen B, van Grunsven LA. P311 modulates hepatic stellate cells migration. Liver Int. 2015;35(4):1253-64. doi: 10.1111/liv.12691. PubMed PMID: 25243526.
