# Supplementary material for: Macrophage Depletion Attenuates Extracellular Matrix Deposition and Ductular Reaction in a Mouse Model of Chronic Cholangiopathies
Source: PLoS One. 2016 Sep 12;11(9):e0162286. doi: 10.1371/journal.pone.0162286 (PMC5019458; doi:10.1371/journal.pone.0162286)
Supplement: S1 Table — (PDF) [file pone.0162286.s010.pdf]

Antibody	Species	Manufacturer	Catalog number	Dilution	Application
**CK19**	Rat monoclonal IgG2a	DSHB	TROMA-III	1:50-1:500	IHC/IF
**F4/80**	Rat monoclonal IgG2b*	ABD Serotec	MCA497GA	1:200	IHC
**Ki67**	Rabbit monoclonal IgG	Thermo Scientific	RM9106	1:200	IF
**αSMA**	Rabbit monoclonal	AbCam	E184	1:200	IHC
**Laminin**	Rabbit	Dako	Z0097	1:4000	IHC/IF
**Alexa Fluor 488**	Donkey Anti-rabbit IgG (H+L)	Thermo Scientific	A21206	1:200	IF
**Alexa Fluor 633**	Goat Anti-rat IgG (H+L)	Thermo Fisher	A11006	1:200	IF
**F4/80 Alexa fluor 678**	Rat Anti-mouse	Life Technology	MF48021		FACS
**EpCAM APC**	Rat Anti-mouse	Biolegend	118214		FACS
**CD32 PE**	Mouse Anti-human	Life Technology	CD3204		FACS
**CD45 FITC**	Rat Anti-mouse	eBioscience	11045185		FACS

Supplementary Table S1. Antibodies used for immunofluorescence (IF), immunohistochemistry (IHC) or FACS.
