# Supplementary material for: Macrophage Depletion Attenuates Extracellular Matrix Deposition and Ductular Reaction in a Mouse Model of Chronic Cholangiopathies
Source: PLoS One. 2016 Sep 12;11(9):e0162286. doi: 10.1371/journal.pone.0162286 (PMC5019458; doi:10.1371/journal.pone.0162286)
Supplement: S2 Table — (PDF) [file pone.0162286.s011.pdf]

Gene	NM	Primer sequences (Fwd./Rev.)	Expression in the liver
**Acta2**	007392.3	CCAGCACCATGAAGATCAAG<br>TGGAAGGTAGACAGCGAAGC	Enhanced expression in activated myofibroblasts/HSCs[2]
**CK19**	008471.2	ATGAGATCATGGCCGAGAAG<br>GGTGTTCAGCTCCTCAATCC	Marker of biliary epithelial cells and liver progenitor cells[3]
**F4/80**	010130.4	CCTGGACGAATCCTGTGAAG<br>GGTGGGACCACAGAGAGTTG	Marker of hepatic macrophages[4]
**Col1a1**	007742.4	ACCTAAGGGTACCGCTGGA<br>TCCAGCTTCTCCATCTTTGC	Enhanced expression in activated myofibroblasts/HSCs[2]
**Gapdh**	008084.2	CCTGCTTCACCACCTTCTTG<br>TGTCCGTCGTGGATCTGAC	Used as reference gene
**Lamc1**	010683.2	TTTGATAGACGCGTGAACGATAA<br>TGGCGGAATTCTCCTTAGA	Enhanced in fibrotic livers[5, 6]
**Cyp3a11**	007818.3	TGAATATGAAACTTGCTCTCACTAAAA<br>CCTTGTCTGCTTAATTTCAGAGGT	Marker of hepatocytes[7]
**Stab2**	138673.2	AGCTGCTGCCTTTAATCCTCA<br>ACTCCGTCTTGATGGTTAGAGTA	Markers for liver sinusoidal cells[8, 9]
**Epcam**	008532.2	GCGGCTCAGAGAGACTGTG<br>CCAAGCATTTAGACGCCAGTTT	Marker of biliary epithelial cells and liver progenitor cells[10]

Supplementary Table S2: Oligonucleotides used for RT-qPCR.

2. Mannaerts I, Leite SB, Verhulst S, Claerhout S, Eysackers N, Thoen LF, et al. The Hippo pathway effector YAP controls mouse hepatic stellate cell activation. J Hepatol. 2015;63(3):679-88. doi: 10.1016/j.jhep.2015.04.011. PubMed PMID: 25908270.
3. Yimlamai D, Christodoulou C, Galli GG, Yanger K, Pepe-Mooney B, Gurung B, et al. Hippo pathway activity influences liver cell fate. Cell. 2014;157(6):1324-38. doi: 10.1016/j.cell.2014.03.060. PubMed PMID: 24906150; PubMed Central PMCID: PMCPMC4136468.
4. Lee SH, Starkey PM, Gordon S. Quantitative-Analysis of Total Macrophage Content in Adult-Mouse Tissues - Immunochemical Studies with Monoclonal-Antibody F4/80. Journal of Experimental Medicine. 1985;161(3):475-89. doi: DOI 10.1084/jem.161.3.475. PubMed PMID: WOS:A1985ACS5200004.
5. Kallis YN, Robson AJ, Fallowfield JA, Thomas HC, Alison MR, Wright NA, et al. Remodelling of extracellular matrix is a requirement for the hepatic progenitor cell response. Gut. 2011;60(4):525-33. doi: 10.1136/gut.2010.224436. PubMed PMID: 21106552.
6. Knittel T, Janneck T, Muller L, Fellmer P, Ramadori G. Transforming growth factor beta 1-regulated gene expression of Ito cells. Hepatology. 1996;24(2):352-60. doi: 10.1053/jhep.1996.v24.pm0008690404. PubMed PMID: 8690404.
7. Anzai K, Chikada H, Tsuruya K, Ida K, Kagawa T, Inagaki Y, et al. Foetal hepatic progenitor cells assume a cholangiocytic cell phenotype during two-dimensional pre-culture. Sci Rep. 2016;6:28283. doi: 10.1038/srep28283. PubMed PMID: 27335264; PubMed Central PMCID: PMCPMC4917868.
8. Hansen B, Longati P, Elvevold K, Nedredal GI, Schledzewski K, Olsen R, et al. Stabilin-1 and stabilin-2 are both directed into the early endocytic pathway in hepatic sinusoidal endothelium via interactions with clathrin/AP-2, independent of ligand binding. Exp Cell Res. 2005;303(1):160-73. doi: 10.1016/j.yexcr.2004.09.017. PubMed PMID: 15572036.
9. Nonaka H, Sugano S, Miyajima A. Serial analysis of gene expression in sinusoidal endothelial cells from normal and injured mouse liver. Biochem Biophys Res Commun. 2004;324(1):15-24. doi: 10.1016/j.bbrc.2004.09.014. PubMed PMID: 15464976.
10. Lu WY, Bird TG, Boulter L, Tsuchiya A, Cole AM, Hay T, et al. Hepatic progenitor cells of biliary origin with liver repopulation capacity. Nat Cell Biol. 2015;17(8):971-83. doi: 10.1038/ncb3203. PubMed PMID: 26192438; PubMed Central PMCID: PMCPMC4612439.
